# Supplementary material for: Neuromuscular Characteristics of Unilateral and Bilateral Maximal Voluntary Isometric Contractions following ACL Reconstruction
Source: Biology (Basel). 2023 Aug 26;12(9):1173. doi: 10.3390/biology12091173 (PMC10525486; doi:10.3390/biology12091173)
Supplement: Supplementary file 1 [file biology-12-01173-s001.zip › biology-2452767-supplementary.pdf]

# Neuromuscular Characteristics of Unilateral and Bilateral Maximal Voluntary Isometric Contractions following ACL Reconstruction

Riccardo Di Giminiani [1,*], Stefano Marinelli[1], Stefano La Greca[1], Andrea Di Blasio[2], Massimo Angelozzi[3], Angelo Cacchio[3]

1. Department of Biotechnological and Applied Clinical Sciences, University of L'Aquila, Italy
2. Department of Medicine and Aging Sciences 'G. D'Annunzio' University of Chieti-Pescara, Italy
3. Department of Life, Health and Environmental Sciences, University of L'Aquila, Italy

## Supplementary Materials

### List of content:

- **Table S1** ICCs (95% confidence limit, lower limit-LL, upper limit-UL) of MVICs and RFD values.
- **Table S2** ICCs (95% confidence limit, lower limit-LL, upper limit-UL) of sEMG values recorded during unilateral MVICs and RFD
- **Table S3** ICCs (95% confidence limit, lower limit-LL, upper limit-UL) of sEMG values recorded during bilateral MVICs.

**Table S1.** ICCs (95% confidence limit, lower limit-LL, upper limit-UL) of MVICs and RFD values.

| | | UL | | | | BL |
|---|---|---|---|---|---|---|
| **Group** | **Variable** | ***Operated Leg*** | ***Non-Operated Leg*** | ***RL*** | ***LL*** | |
| **ACL** | **MVICs** | 0.95 (0.85-0.98) | 0.98 (0.93-0.99) | *NA* | *NA* | 0.99 (0.98-1) |
| | **RFD** | 0.88 (0.67-0.96) | 0.83 (0.48-0.93) | *NA* | *NA* | 0.87 (0.65-0.96) |
| **CG** | **MIVCs** | *NA* | *NA* | 0.99 (0.95-1) | 0.83 (0.40-0.96) | 0.94 (0.82-0.98) |
| | **RFD** | *NA* | *NA* | 0.92 (0.77-0.97) | 0.85 (0.59-0.95) | 0.95 (0.84-0.98) |

Abbreviations: unilateral (UL), bilateral (BL), maximal voluntary isometric contractions (MVICs), rate of force development (RFD), right leg (RL), and left leg (LL)

**Table S2.** ICCs (95% confidence limit, lower limit-LL, upper limit-UL) of sEMG values recorded during unilateral MVICs and RFD.

| Variable | ACL Group | | Control Group | |
|---|---|---|---|---|
| | Unilateral Exercise | | *Unilateral Exercise* | |
| | *Operated Leg* | *Non-Operated Leg* | *Right Leg* | *Left Leg* |
| **$sEMG_{RMS}$ relative to MVICs** | | | | |
| **VL** | 0.91 (0.77-0.97) | 0.95 (0.87-0.98) | 0.88 (0.66-0.96) | 0.94 (0.82-0.98) |
| **VM** | 0.74 (0.43-0.91) | 0.91 (0.74-0.97) | 0.91 (0.75-0.97) | 0.92 (0.78-0.98) |
| **RF** | 0.94 (0.83-0.98) | 0.93 (0.78-0.98) | 0.93 (0.79-0.98) | 0.98 (0.93-0.99) |
| **BF** | 0.94 (0.80-0.98) | 0.80 (0.48-0.93) | 0.98 (0.94-0.99) | 0.90 (0.71-0.97) |
| | | | | |
| **$sEMG_{RMS}$ relative to RFD** | | | | |
| **VL** | 0.89 (0.57-0.87) | 0.98 (0.93-0.99) | 0.95 (0.85-0.98) | 0.89 (0.68-0.96) |
| **VM** | 0.88 (0.53-0.93) | 0.91 (0.75-0.97) | 0.91 (0.75-0.97) | 0.89 (0.70-0.97) |
| **RF** | 0.87 (0.59-0.96) | 0.84 (0.55-0.95) | 0.80 (0.49-0.93) | 0.80 (0.48-0.93) |
| **BF** | 0.72 (0.39-0.90) | 0.87 (0.65-0.96) | 0.95 (0.86-0.99) | 0.85 (0.60-0.95) |

Abbreviations: maximal voluntary isometric contractions (MVICs), vastus lateralis (VL), vastus medialis (VM), rectus femoris (RF), biceps femoris (BF) and rate of force development (RFD).

**Table S3.** ICCs (95% confidence limit, lower limit-LL, upper limit-UL) of sEMG values recorded during bilateral MVICs.

| **Variable** | **ACL Group** | | **Control Group** | |
|---|---|---|---|---|
| | *Bilateral Exercise* | | *Bilateral Exercise* | |
| | *Operated Leg* | *Non-Operated Leg* | *Right Leg* | *Left Leg* |
| **$sEMG_{RMS}$ relative to MVICs** | | | | |
| **VL** | 0.98 (0.93-0.99) | 0.78 (0.44-0.93) | 0.87 (0.65-0.96) | 0.97 (0.90-0.99) |
| **VM** | 0.93 (0.79-0.98) | 0.88 (0.77-0.96) | 0.93 (0.80-0.98) | 0.97 (0.90-0.99) |
| **RF** | 0.81 (0.47-0.94) | 0.98 (0.94-0.99) | 0.75 (0.38-0.92) | 0.92 (0.77-0.97) |
| **BF** | 0.94 (0.83-0.98) | 0.98 (0.93-0.99) | 0.98 (0.93-0.99) | 0.92 (0.78-0.98) |
| | | | | |
| **$sEMG_{RMS}$ relative to RFD** | | | | |
| **VL** | 0.93 (0.80-0.98) | 0.87 (0.63-0.96) | 0.93 (0.79-0.98) | 0.91 (0.73-0.97) |
| **VM** | 0.89 (0.70-0.97) | 0.92 (0.77-0.97) | 0.94 (0.83-0.98) | 0.96 (0.87-0.99) |
| **RF** | 0.89 (0.68-0.97) | 0.95 (0.84-0.99) | 0.81 (0.51-0.94) | 0.81 (0.49-0.93) |
| **BF** | 0.89 (0.60-0.97) | 0.91 (0.72-0.97) | 0.81 (0.51-0.94) | 0.88 (0.67-0.96) |

Abbreviations: maximal voluntary isometric contractions (MVICs), vastus lateralis (VL), vastus medialis (VM), rectus femoris (RF), biceps femoris (BF) and rate of force development (RFD).
